# Revealing genome-scale transcriptional regulatory landscape of OmpR highlights its expanded regulatory roles under osmotic stress in *Escherichia coli* K-12 MG1655

**DOI:** 10.1038/s41598-017-02110-7

**Published:** 2017-05-19

**Authors:** Sang Woo Seo, Ye Gao, Donghyuk Kim, Richard Szubin, Jina Yang, Byung-Kwan Cho, Bernhard O. Palsson

**Affiliations:** 1 School of Chemical and Biological Engineering and Institute of Chemical Process, Seoul National University, 1 Gwanak-ro, Gwanak-Gu, Seoul 08826 Republic of Korea; 2 Department of Bioengineering, University of California San Diego, La Jolla, CA 92093 USA; 3 Division of Biological Science, University of California San Diego, La Jolla, CA 92093 USA; 4 Department of Pediatrics, University of California San Diego, La Jolla, CA 92093 USA; 5 Department of Genetic Engineering, College of Life Sciences, Kyung Hee University, Yongin, 446-701 Republic of Korea; 6 Department of Biological Sciences, Korea Advanced Institute of Science and Technology, Daejeon, 305-701 Republic of Korea; 7 Novo Nordisk Foundation Center for Biosustainability, Technical University of Denmark, 2800 Lyngby, Denmark

## Abstract

A transcription factor (TF), OmpR, plays a critical role in transcriptional regulation of the osmotic stress response in bacteria. Here, we reveal a genome-scale OmpR regulon in *Escherichia coli* K-12 MG1655. Integrative data analysis reveals that a total of 37 genes in 24 transcription units (TUs) belong to OmpR regulon. Among them, 26 genes show more than two-fold changes in expression level in an OmpR knock-out strain. Specifically, we find that: 1) OmpR regulates mostly membrane-located gene products involved in diverse fundamental biological processes, such as *narU* (encoding nitrate/nitrite transporter), *ompX* (encoding outer membrane protein X), and *nuoN* (encoding NADH:ubiquinone oxidoreductase); 2) by investigating co-regulation of entire sets of genes regulated by other stress-response TFs, stresses are surprisingly independently regulated among each other; and, 3) a detailed investigation of the physiological roles of the newly discovered OmpR regulon genes reveals that activation of *narU* represents a novel strategy to significantly improve osmotic stress tolerance of *E*. *coli*. Thus, the genome-scale approach to elucidating regulons comprehensively identifies regulated genes and leads to fundamental discoveries related to stress responses.

## Introduction

The key to bacteria’s survival is their ability to sense and respond to the different environmental stresses to which they are exposed. OmpR coordinates genome-scale transcriptional responses to osmotic stress with its cognate sensor EnvZ in many enteric bacteria^1^. The general role of this TF has been investigated using *in vitro* DNA-binding experiments and comparative transcriptomics^2–17^. For example, OmpR controls production of two outer membrane porins, OmpF and OmpC, that enable bacterial cells to survive fluctuations in the osmolarity of the growth medium^12, 13, 15, 16^. However, much less is known about *in vivo* OmpR-binding at the genome-scale under osmotic stress and the regulon that they comprise in *E*. *coli* K-12 MG1655. There were several reports that investigated the genome-scale role of OmpR in *Salmonella enterica* and *Salmonella Typhimurium*, and these studies found some novel OmpR-regulated operons that are part of the *S*. *enterica* ancillary genome^18, 19^. Also, there was a study that investigated the response of acid stress by OmpR in *E*. *coli* K-12 CSH50 and compared it with that of *S*. *Typhimurium* ^19^. An earlier study also had focused on the transcriptome response of acid stress by OmpR in *E*. *coli* K-12 BW25113^20^.

It is expected that a complete reconstruction of the OmpR transcriptional regulatory network (TRN) in response to osmotic stress will reveal a more detailed understanding of its regulation on a genome-scale even in *E*. *coli* K-12 MG1655. A better understanding of the OmpR regulatory network can shed further light on its functions if it also coordinates stress responses with other fundamental cellular/metabolic processes as do other TFs^21–23^. Based on such elucidation, inter-relationships among other regulons such as those related to different environmental stress responses can be investigated^21–23^. A systems biology approach based on genome-scale experimental measurements and integrative analysis can contribute to such an understanding.

Here, a systems biology approach was applied to decipher the OmpR regulatory networks under osmotic stress (NaCl treatment). We first examine the OmpR-binding sites on the *E*. *coli* K-12 MG1655 genome from chromatin immunoprecipitation with lambda exonuclease digestion followed by high-throughput sequencing (ChIP-exo). Then, we measure transcription levels of genes in wild-type and knock-out mutant of OmpR on a genome-scale from strand-specific massively parallel cDNA sequencing (RNA-seq). These datasets are integrated to identify causal relationships and reconstruct the OmpR regulon. A combination of topological and functional analyses of the OmpR regulon provides an unprecedented and comprehensive view of genome-wide regulatory roles of this TF under osmotic stress.

## Results and Discussion

### Genome-wide binding profile of OmpR under osmotic stress

**Figure 1:**
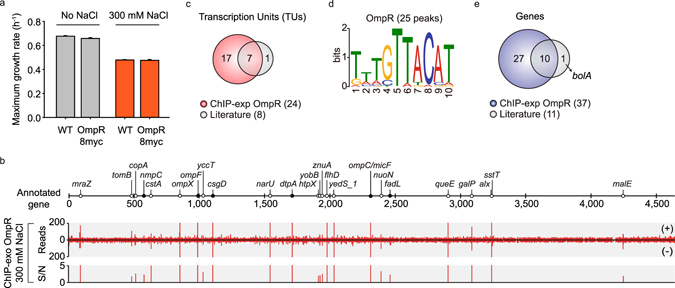
Genome-wide profiles of OmpR-binding sites. (**a**) Effect of myc-tag of OmpR on cellular growth in response to osmotic stress (0.3 M NaCl) (**b**). An overview of OmpR-binding profiles across the *E*. *coli* genome at mid-exponential growth phase under osmotic stress condition. Black and white dots indicate previously known and newly found OmpR-binding sites, respectively. S/N denotes signal to noise ratio. (+) and (−) indicate forward and reverse reads, respectively. Binding peaks that overlap with Mock-IP signal were eliminated. (**c**,**d**) Comparison of genome-wide OmpR-binding sites obtained from this study (ChIP-exo) under osmotic stress with the known binding sites from the literature in terms of (**c**) transcription units and (**d**) genes. (**e**) Sequence motif logo represents the DNA binding motifs of OmpR from 25 binding peaks.

Over the last two decades, 8 binding sites (TUs) have been identified for OmpR in *E*. *coli* by *in vitro* DNA-binding experiments and relevant mutational analysis^2–17^. However, direct measurements of genome-wide *in vivo* OmpR binding are not available in *E*. *coli* K-12 MG1655. Thus, we first performed a ChIP-exo experiment to determine *in vivo* genome-scale binding profiles of OmpR in *E*. *coli* K-12 MG1655 under osmotic stress condition (0.3 M NaCl treatment). We used the 8-myc epitope tagging approach as previously reported^21^ to perform a ChIP-exo experiment, and epitope tagging did not change the ability of *E*. *coli* to respond to osmotic stress (Fig. 1a). From ChIP-exo experiments, we identified 25 reproducible binding sites for OmpR under osmotic stress (Fig. 1b and Supplementary Table 1). All these binding sites were found within regulatory regions including upstream of promoters, promoters, and 5′-proximal to coding regions (Supplementary Table 1). These results show a strong preference of OmpR-binding site locations being within the noncoding intergenic regions similar to other transcription regulators^21–27^. We also detected 88% (7 of 8) of OmpR-binding sites reported from previous studies (Fig. 1c and Supplementary Table 2). The only exception was the upstream region of *bolA* but it is unclear why this site is missing from the dataset obtained here. Nevertheless, we significantly expanded the current knowledge of OmpR binding information on the genome.

To identify DNA sequence motifs of OmpR binding sites, we first used the MEME software tool^28^ with the genomic sequences of binding sites. The identified sequence motif of OmpR from 25 binding peaks (KWWGTTACAT) was consistent with the previously characterized repeat sequence of the OmpR-binding site (tTTGTTACAT) (Fig. 1e)^2^. In addition, we used two other methods, ChIPMunk and diChIPMunk, to identify DNA sequence motifs of OmpR^29–31^. The identified sequence motifs were similar to that from the MEME software tool in that these two methods generated the previously known repeat sequence (GTTACATTTTGTTACAT) (Supplementary Fig. 1). We believe that the OmpR-binding sites revealed here are *bona fide* binding sites given the consistency of the known binding sites and their motifs.

### Genome-wide reconstruction of the OmpR regulon

**Figure 2:**
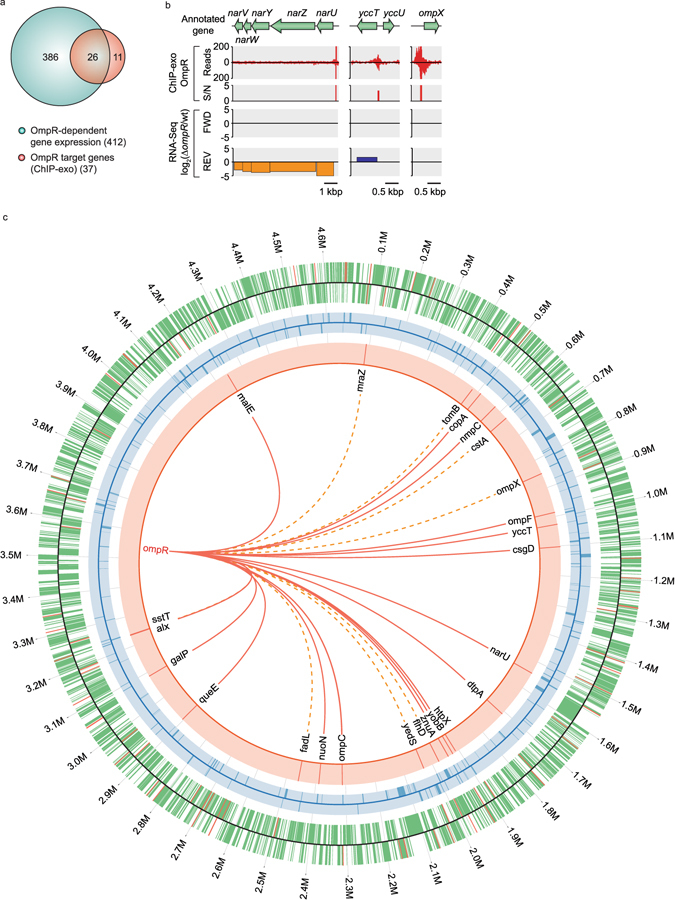
Regulatory causation in OmpR regulon. (**a**) Causal relationships between direct associations of OmpR and changes in transcript levels of genes. (**b**) Regulatory causation of individual ORFs governed by OmpR in response to osmotic stress. Examples of activation (*narUZYWV*), repression (*yccT*), and not determined (no change in transcript level, *ompX*). S/N denotes signal to noise ratio. FWD and REV indicate log 2 relative transcript levels from forward and reverse strands, respectively. (**c**) Genome-wide map of OmpR-binding profile was overlaid with differential transcriptional response to OmpR knockout under osmotic stress. Red, blue, and green denote ChIP-exo, RNA-seq, and annotation, respectively. Solid lines indicate genes with OmpR binding with changes in expression level upon OmpR knock-out. Dotted lines indicate the genes with TF bindings but without changes in expression level upon OmpR knockout. Only the first gene of the operon was shown. The figure was made using Circos version 0.67.

Before this study, a total of 11 genes in 8 transcription units (TUs) had been characterized as members of OmpR regulons. The ChIP-exo datasets of this study expanded the size of the OmpR regulons to contain 37 target genes in 24 TUs (Fig. 1d and Supplementary Table 1). To determine the causal relationship between the genome-wide binding of OmpR and changes in levels of RNA transcripts of regulon genes, transcript levels of wild-type and those of a deletion mutant (Δ*ompR*) were compared with both grown under stress condition with 0.3 M NaCl treatment. Overall, a total of 412 genes were differentially expressed (log_2_ fold change ≥ 1 and false discovery rate (FDR) ≤ 0.01) (Fig. 2a and Supplementary Data 1).

Combining the genome-wide OmpR-binding map with the OmpR-dependent transcriptome enabled us to define the causality between the binding of OmpR and transcript levels of the corresponding genes under osmotic stress. Of the 37 target genes discovered from the ChIP-exo experiment, 26 genes in 16 TUs were directly regulated by OmpR under osmotic stress (Fig. 2a and Supplementary Table 1). For example, the promoter region of *narUZYWV* TU was extensively occupied by OmpR under the osmotic stress condition and this binding significantly increased transcript levels under this condition (Fig. 2b, left panel). A total of 14 genes were regulated by this regulatory mode, and half of them were previously identified as regulon members (Supplementary Table 1).

In contrast, association of OmpR on the *yccT* TU decreased transcript level under the same condition (Fig. 2b, middle panel). We identified a total of 12 genes repressed by binding of OmpR and only one of them (*nmpC*) was previously characterized (Supplementary Table 1). For the remaining 11 genes, we were not able to find statistically significant changes in expression levels. However, reproducible binding peaks and motifs were detected at the regulatory regions of these genes just like the *ompX* case (Fig. 2b, right panel). It may be possible that other co-regulating TFs partially take over the regulatory role on each gene under our experimental stress condition when OmpR is deleted on the genome. The calculation of the relative distance from binding centers of OmpR to transcription start site (TSS)^32, 33^ showed that it binds to the sites either upstream or downstream of the corresponding promoter region, whether OmpR can act as a repressor or an activator (Supplementary Table 1). Like other TFs, OmpR may prevent binding of other repressors or directly activate transcription by recruiting RNAP, or exclude RNAP binding at this region to repress transcription. Based on these results, we graphically summarized the transcriptional regulatory network of OmpR by combining ChIP-exo and RNA-seq data on *E*. *coli* genome (Fig. 2c).

### Interaction of OmpR regulon with other stress-response TFs

**Figure 3:**
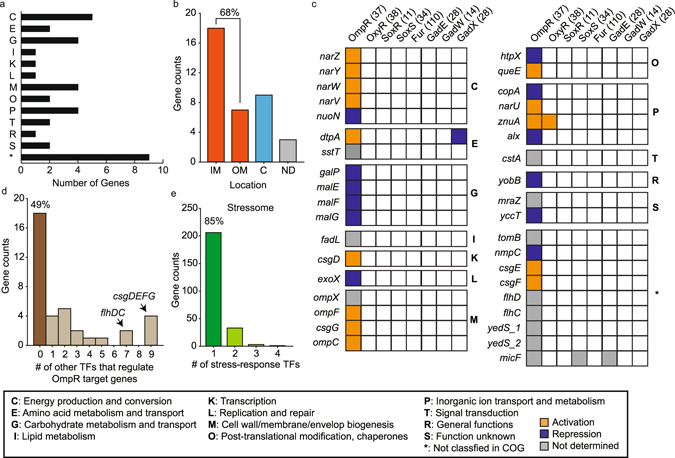
Functional delineation of OmpR regulon and its interaction with other TFs. (**a**) Functional classification (COG) of OmpR regulon. (**b**) Distribution of functional location of gene products of OmpR regulon. IM; inner membrane, OM; outer membrane, C; cytosol, ND; not determined. (**c**) Integration of the OmpR regulon information (37 target genes) with other publicly available regulon information of stress-responsive TFs for oxidative stress (OxyR, SoxR, and SoxS), iron imbalance stress (Fur), and acid stress (GadE, GadW, and GadX). The 37 target genes were categorized into 13 groups based on their COG functional annotations. The amber and dark blue boxes indicate activation and repression of transcription by direct association of OmpR, respectively. The grey box indicates that the transcription regulation could not be determined though the direct association of OmpR. (**d**) The distribution of the number of OmpR target genes regulated by other TFs among the entire set of TFs. (**e**) The distribution of 243 genes consisted of regulon members of 8 different stress-response TFs according to the number of stress-response TFs regulating those genes.

Next, we classified 37 target genes based on clusters of orthologous groups (COG) protein database (Fig. 3a)^34^. These genes were categorized into 13 different COG functional categories including energy production and conversion (C), carbohydrate metabolism and transport (G), cell wall/membrane/envelope biogenesis (M), and inorganic ion transport and metabolism (P). Although their functional categories were diverse, 68% of these gene products (25 of 37) were known to be located in either inner (18) or outer (7) membranes (Fig. 3b)^33^. Only 9 gene products were located in cytosol and 3 gene products were unknown. These results indicate that OmpR mostly regulates membrane proteins of various functional processes and thus affects the composition of the membrane proteomes.

We further integrated the information from the OmpR regulon with other regulon data of TFs related to oxidative stress (OxyR, SoxR, and SoxS), iron imbalance stress (Fur), and acid stress (GadE, GadW, and GadX) based on our previous studies^21–23^ (Fig. 3c). Surprisingly, 92% (34 of 37) of OmpR regulon members were not regulated by any of those 7 TFs. The exceptions were *dtpA* encoding tripeptide/dipeptide: H^+^ symporter (regulated by GadX), *znuA* encoding Zn^2+^ ABC transporter (regulated by OxyR), and *micF* encoding small regulatory RNA (regulated by SoxS and GadE).

We continued our analysis by including the entire list of TFs of *E*. *coli*. Still, 49% (18 of 37) of the regulon members did not have any known regulatory action by any other TF (Fig. 3d and Supplementary Table 3). Two TUs, *flhDC* and *csgDEFG*, were regulated by an additional 7 and 9 other TFs, respectively, indicating the importance of precise regulation of cell motility in various environments.

In addition, by combining our previous results on reconstruction of TRNs on oxidative stress, iron imbalance stress, and acid stress with this study, we could make a list of genes consisting of entire sets of gene products regulated by corresponding 8 different TFs in response to these four different environmental stresses (Supplementary Data 2). A total of 243 different genes were regulated by these TFs, and surprisingly 85% (206 of 243) of these genes were only regulated by a single TF (Fig. 3e and Supplementary Data 2). Then, 13% (33 of 243) of these genes were regulated by two TFs. Based on these results, it appeared that these genes tend to be regulated by each TF separately depending on the types of stress with marginal overlap between regulons, while the sigma factor networks appear to provide the cell with regulatory redundancies^35^.

### Physiological roles and evolutionary aspects of the OmpR regulon

**Figure 4:**
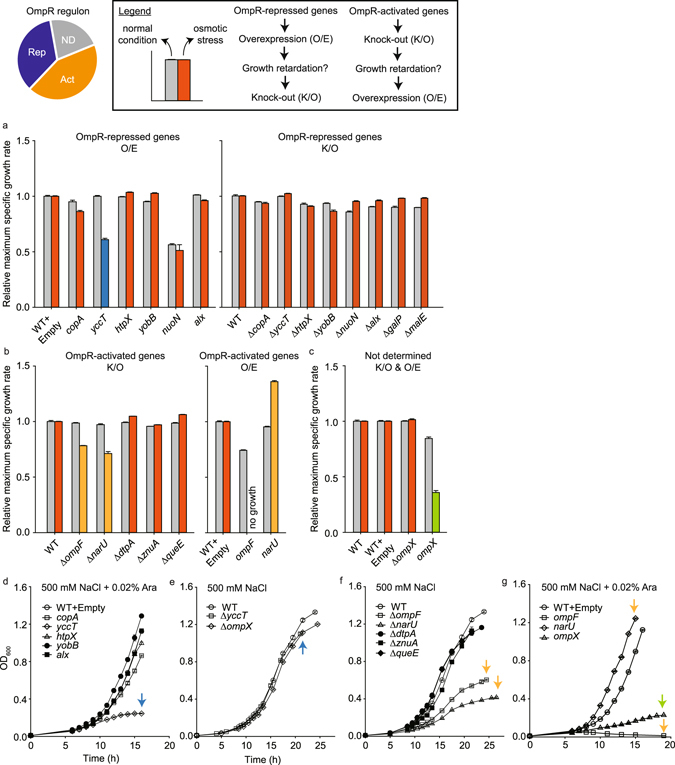
Examination of physiological role of OmpR regulon under osmotic stress. (**a**) OmpR-repressed genes were overexpressed (O/E) to investigate their effects on cellular growth rate under normal and osmotic stress conditions. If mutant strains showed retarded growth rate under osmotic stress conditions, the corresponding genes were knocked-out (K/O) and growth rates of mutant strains were measured. In order to eliminate any potential caveat from differential expression of membrane-bound proteins, we additionally constructed mutant strains of OmpR-repressed genes. The same approach was applied to (**b**) OmpR-activated genes and (**c**) Not-determined genes. The relative maximum specific growth rates were represented as normalized by those of wild-type strains under each condition. Time-course growth curves (OD at 600 nm) of mutants were depicted depending on (**d**,**g**) overexpression and (**e**,**f**) knock-out conditions. For the osmotic stress condition, the growth media were supplemented with 500 mM NaCl. The mean and standard deviation from independent biological triplicate were represented.

In order to further examine physiological roles of the OmpR regulon identified above, we examined effects of single gene knock-out or overexpression on cell growth under osmotic stress. We hypothesized that if any OmpR-repressed gene is physiologically important for osmotic stress tolerance, overexpression of this gene may result in growth retardation. When we overexpressed 6 newly identified genes (*copA*, *yccT*, *htpX*, *yobB*, *nuoN*, and *alx*) among OmpR-repressed genes and compared their growth rates with that of wild-type under both normal and osmotic stress conditions, only overexpression of *yccT* showed 40% decrease in growth rate under osmotic stress (Fig. 4a,d). Interestingly, overexpression of *nuoN* encoding NADH:ubiquinone oxidoreductase retarded growth of cells even under normal conditions (Fig. 4a). These results show that overexpression of membrane-associated proteins had a negative effect on *E*. *coli* growth under the experimental osmotic stress and protein induction conditions. Thus, in order to eliminate any potential caveats related to differential protein expression, we additionally examined whether deletion of these OmpR-repressed genes would improve the tolerance of cells toward osmotic stress. However, all mutant strains showed similar growth rates under both normal and osmotic stress conditions compared to that of wild-type (Fig. 4a,e).

Based on a similar hypothesis, we deleted 3 newly identified and 2 previously known genes (*ompF*, *narU*, *dtpA*, *znuA*, and *queE*) among OmpR-activated genes. In this case, deletion of *ompF* encoding outer membrane porin F and *narU* encoding nitrate/nitrite transporter showed a 21% and 30% decrease in growth rate under osmotic stress, respectively (Fig. 4b,f). Interestingly, overexpression of *ompF* slightly retarded growth of cells under normal conditions but perfectly abolished cellular growth under osmotic stress (Fig. 4b,g). However, the overexpression of *narU* significantly improved cell growth (36%) under osmotic stress (Fig. 4b,g). It is unclear at this point how overexpression of *narU* contributes to the restoration of growth under osmotic stress, but this can be a promising strategy to improve cellular tolerance toward osmotic stress when engineering tolerance into a new strain. We also examined the physiological role of *ompX* (encoding outer membrane protein X) that did not show changes in transcription level when it was deleted but showed strong binding of OmpR. Although deletion of *ompX* did not show any change in growth rate, overexpression of *ompX* showed similar growth retardation to the *ompF* case (Fig. 4c,e,g). Based on our growth tests of mutant strains of membrane proteins (*yccT*, *ompF*, and *ompX*), it appears that the composition of membrane proteins has to be precisely controlled to achieve proper response to osmotic stress.

**Figure 5:**
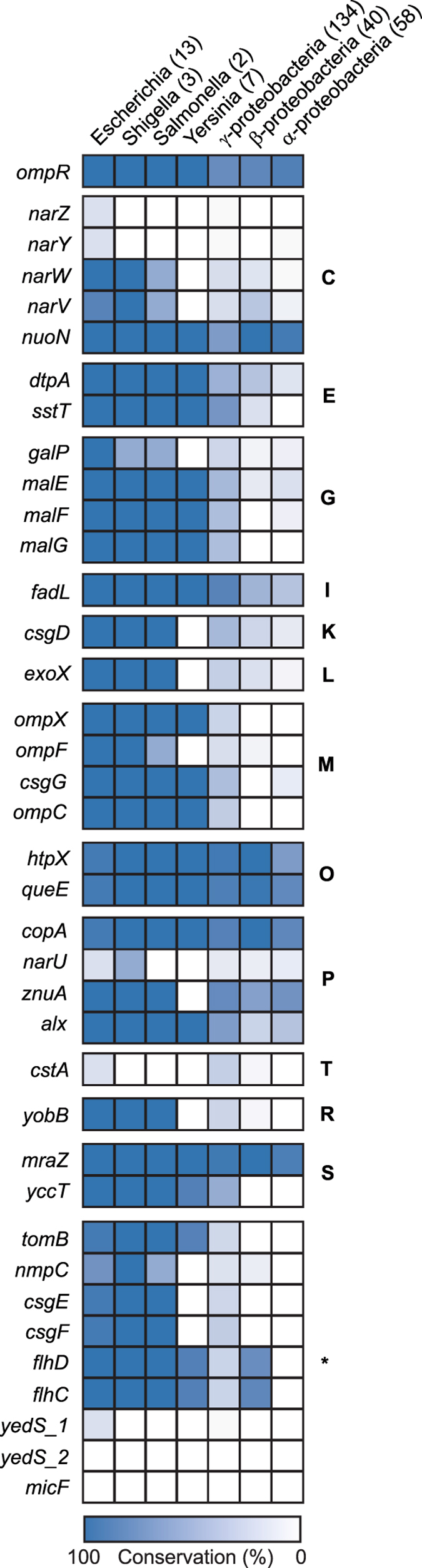
Evolutionary perspective on OmpR regulon. Conservation levels of the OmpR and its regulon genes across Escherichia, Shigella, Salmonella, Yersinia, γ-, β-, and α-proteobacteria are illustrated by the ortholog calculation. The genes were divided into 13 groups by their functions as in Fig. 3.

Since osmotic stress response is important for the survival of other bacteria, we examined the phylogenetic distribution of OmpR and its target genes identified here. We determined how the *E*. *coli* K-12 MG1655 OmpR regulon is conserved within proteobacteria by comparing gene orthologues in 13 Escherichia, 3 Shigella, 2 Salmonella, 7 Yersinia, 134 γ-proteobacteria, 40 β-proteobacteria, and 58 α-proteobacteria (Fig. 5). Interestingly, OmpR was highly conserved across species, but genes in the regulon of *E*. *coli* K-12 MG1655 were not. This result suggests that other species may have different regulatory networks to cope with osmotic stress. For example, the *narUZYWV* operon was poorly conserved even in Escherichia, indicating that activation of this operon under osmotic stress might be a unique strategy of *E*. *coli* K-12 MG1655 to improve the cell’s growth under osmotic stress.

In summary, we have integrated disparate data types from genome-scale experimental methods and analyzed them to describe how this system biology approach guided us to comprehensively understand the fundamental role of the OmpR regulon in response to osmotic stress in *E*. *coli* K-12 MG1655. We found that OmpR tends to regulate gene products located in membranes among various biological processes. Detailed examination of physiological roles of OmpR regulon members revealed that activation of *narU* was important to achieve tolerance toward osmotic stress and precise control of the composition of OmpR-regulated membrane proteins is crucial for the cell’s viability. In addition to previous reconstruction studies, the expanded OmpR regulatory network under osmotic stress can be integrated into genome-scale computational models for better predictive capability of cellular stress response^36^.

## Methods

### Bacterial strains, primers, media, and growth conditions

The OmpR-8myc *E*. *coli* strain was generated by a λ Red-mediated site-specific recombination system targeting the C-terminal region^37^. Deletion mutants including *ompR* and other regulon members were also constructed by a λ Red-mediated site-specific recombination system^38^. Overexpression mutants were constructed by using directional pBAD plasmid system (Invitrogen) according to the manufacturer’s instructions. All primers used for the construction of plasmids and strains are presented in Supplementary Table 4. For overnight culture, *E*. *coli* strains were inoculated into fresh 70 ml of 0.2% (w/v) glucose M9 minimal medium in 500 ml flasks at 37 °C with 250 rpm. To make osmotic stress for ChIP-exo and RNA-seq, the overnight cultures were inoculated to OD_600_ = 0.02 into another fresh 70 ml of M9 minimal medium in 500 ml flasks and were supplemented with 0.3 M sodium chloride (NaCl) at OD_600_ = 0.5 ± 0.04 and incubated for 30 min with stirring. In cases of growth tests under normal and osmotic stress conditions, the same media were used for normal conditions and were supplemented with 0.5 M concentration of NaCl at the beginning of the culture. For overexpression strains, media were also supplemented with 0.02% arabinose and wild-type strain harboring empty plasmid was used as a control.

### ChIP-exo and data processing

To identify the genome-wide OmpR-binding map *in vivo*, the DNA bound to OmpR were isolated by chromatin immunoprecipitation (ChIP) with the antibodies recognizing myc tag (9E10, Santa Cruz Biotechnology) and Dynabeads Pan Mouse IgG magnetic beads (Invitrogen) followed by multiple stringent washing steps^21^. Chromatin-beads materials were used to perform on-bead enzymatic reactions of the original ChIP-exo method with our previous modifications^39, 40^ to construct sequencing libraries. We sequenced these DNA libraries using in-house MiSeq machine (Illumina) following the manufacturer’s instructions. ChIP-exo experiments were performed in biological duplicate. Sequence reads obtained from ChIP-exo experiments were mapped onto the *E*. *coli* reference genome (NC_000913.2) using bowtie^41^ with default options in order to generate SAM output files. Previously published MACE program (https://code.google.com/p/chip-exo/)^42^ was used to write an in-house script to define peak candidates from independent biological duplicates with sequence depth normalization. To remove false-positive peaks, peaks having signal-to-noise (S/N) ratio less than 1.0 or having signals on the same position with Mock-IP were not considered^39, 40^.

### RNA-seq and data processing

We isolated total RNA including small RNAs using Qiagen RNeasy Plus Mini Kit (Qiagen) by treating cells with RNAprotect Bacteria Reagent (Qiagen) in accordance with the manufacturer’s instructions. Strand-specific dUTP RNA-seq^43^ was performed with the modifications^39, 40^ to build sequencing libraries in independent biological duplicate. The library samples were sequenced using in-house MiSeq machine (Illumina) in accordance with the manufacturer’s instructions. Sequence reads obtained from RNA-seq experiments were mapped onto the same reference genome (NC_000913.2) using bowtie^41^ with the maximum insert size of 1000 bp, and 2 maximum mismatches after trimming 3 bp at 3′ ends. These files were used for Cufflinks^42^ and Cuffdiff to calculate fragments per kilobase of exon per million fragments (FPKM) and differential expression, respectively, with default options and library type of dUTP RNA-seq. Genes that showed differential expression with log2 fold change ≥ 1.0 and a false discovery rate (FDR) value ≤ 0.01 from the Cuffdiff output were considered as differentially expressed genes in our study.

### Motif search and analysis

The OmpR-binding motif analysis was first done by using the MEME tool from the MEME software suite with default settings^28^. In addition, we used two other methods, ChIPMunk and diChIPMunk, to identify DNA sequence motifs of OmpR^29–31^.

### Conservation analysis of OmpR regulon

For conservation analysis, annotation information of strains and species were obtained from the SEED server (http://theseed.org). After that, ortholog to *E*. *coli* K-12 MG1655 was found from RAST (Rapid Annotation using Subsystem Technology) server^43^. Conservation level of *ompR* and genes of OmpR regulon were calculated from orthologues retained from RAST output.

## Electronic supplementary material

Supplementary Information

Dataset1, Dataset2

## References

[1] Forst SA, Roberts DL (1994). Signal transduction by the EnvZ-OmpR phosphotransfer system in bacteria. Res. Microbiol..

[2] Tsung K, Brissette RE, Inouye M (1989). Identification of the DNA-binding domain of the OmpR protein required for transcriptional activation of the ompF and ompC genes of *Escherichia coli* by *in vivo* DNA footprinting. J. Biol. Chem..

[3] Coyer J, Andersen J, Forst SA, Inouye M, Delihas N (1990). micF RNA in ompB mutants of Escherichia coli: different pathways regulate micF RNA levels in response to osmolarity and temperature change. J. Bacteriol..

[4] Black PN (1991). Primary sequence of the Escherichia coli fadL gene encoding an outer membrane protein required for long-chain fatty acid transport. J. Bacteriol..

[5] Slauch JM, Silhavy TJ (1991). cis-acting ompF mutations that result in OmpR-dependent constitutive expression. J. Bacteriol..

[6] Coll JL, Heyde M, Portalier R (1994). Expression of the nmpC gene of Escherichia coli K-12 is modulated by external pH. Identification of cis-acting regulatory sequences involved in this regulation. Mol. Microbiol..

[7] Huang KJ, Schieberl JL, Igo MM (1994). A distant upstream site involved in the negative regulation of the Escherichia coli ompF gene. J. Bacteriol..

[8] Rampersaud A, Harlocker SL, Inouye M (1994). The OmpR protein of Escherichia coli binds to sites in the ompF promoter region in a hierarchical manner determined by its degree of phosphorylation. J. Biol. Chem..

[9] Yamamoto K (2000). Negative regulation of the bolA1p of Escherichia coli K-12 by the transcription factor OmpR for osmolarity response genes. FEMS Microbiol. Lett..

[10] Prigent-Combaret C (2001). Complex regulatory network controls initial adhesion and biofilm formation in Escherichia coli via regulation of the csgD gene. J. Bacteriol..

[11] Qin L, Yoshida T, Inouye M (2001). The critical role of DNA in the equilibrium between OmpR and phosphorylated OmpR mediated by EnvZ in Escherichia coli. Proc. Natl. Acad. Sci. USA.

[12] Mattison K, Oropeza R, Byers N, Kenney LJ (2002). A phosphorylation site mutant of OmpR reveals different binding conformations at ompF and ompC. J. Mol. Biol..

[13] Oshima T (2002). Transcriptome analysis of all two-component regulatory system mutants of Escherichia coli K-12. Mol. Microbiol..

[14] Goh EB, Siino DF, Igo MM (2004). The Escherichia coli tppB (ydgR) gene represents a new class of OmpR-regulated genes. J. Bacteriol..

[15] Jubelin G (2005). CpxR/OmpR interplay regulates curli gene expression in response to osmolarity in Escherichia coli. J. Bacteriol..

[16] Yoshida T, Qin L, Egger LA, Inouye M (2006). Transcription regulation of ompF and ompC by a single transcription factor, OmpR. J. Biol. Chem..

[17] Ogasawara H, Yamada K, Kori A, Yamamoto K, Ishihama A (2010). Regulation of the Escherichia coli csgD promoter: interplay between five transcription factors. Microbiology.

[18] Perkins TT (2013). ChIP-seq and transcriptome analysis of the OmpR regulon of Salmonella enterica serovars Typhi and Typhimurium reveals accessory genes implicated in host colonization. Mol. Microbiol.

[19] Quinn HJ, Cameron AD, Dorman CJ (2014). Bacterial regulon evolution: distinct responses and roles for the identical OmpR proteins of Salmonella Typhimurium and Escherichia coli in the acid stress response. PLoS Genet..

[20] Stincone A (2011). A systems biology approach sheds new light on Escherichia coli acid resistance. Nucleic Acids Res..

[21] Seo SW (2014). Deciphering Fur transcriptional regulatory network highlights its complex role beyond iron metabolism in Escherichia coli. Nat. Commun..

[22] Seo SW, Kim D, O’Brien EJ, Szubin R, Palsson BO (2015). Decoding genome-wide GadEWX-transcriptional regulatory networks reveals multifaceted cellular responses to acid stress in Escherichia coli. Nat. Commun..

[23] Seo SW, Kim D, Szubin R, Palsson BO (2015). Genome-wide Reconstruction of OxyR and SoxRS Transcriptional Regulatory Networks under Oxidative Stress in Escherichia coli K-12 MG1655. Cell Rep..

[24] Cho BK, Barrett CL, Knight EM, Park YS, Palsson BO (2008). Genome-scale reconstruction of the Lrp regulatory network in Escherichia coli. Proc. Natl. Acad. Sci. USA.

[25] Federowicz S (2014). Determining the control circuitry of redox metabolism at the genome-scale. PLoS Genet..

[26] Cho S (2015). The architecture of ArgR-DNA complexes at the genome-scale in Escherichia coli. Nucleic Acids Res..

[27] Kim JN (2015). Genome-scale analysis reveals a role for NdgR in the thiol oxidative stress response in Streptomyces coelicolor. BMC Genomics.

[28] Bailey TL (2009). MEME SUITE: tools for motif discovery and searching. Nucleic Acids Res..

[29] Kulakovskiy IV, Boeva VA, Favorov AV, Makeev VJ (2010). Deep and wide digging for binding motifs in ChIP-Seq data. Bioinformatics.

[30] Kulakovskiy I (2013). From binding motifs in ChIP-Seq data to improved models of transcription factor binding sites. J. Bioinform. Comput. Biol..

[31] Levitsky VG (2014). Application of experimentally verified transcription factor binding sites models for computational analysis of ChIP-Seq data. BMC Genomics.

[32] Kim D (2012). Comparative analysis of regulatory elements between Escherichia coli and Klebsiella pneumoniae by genome-wide transcription start site profiling. PLoS Genet..

[33] Karp, P. D. *et al*. The EcoCyc Database. *EcoSal Plus* **6**, doi:10.1128/ecosalplus.ESP-0009-2013 (2014). 10.1128/ecosalplus.ESP-0009-2013 PMC4243172 26442933

[34] Galperin MY, Makarova KS, Wolf YI, Koonin EV (2015). Expanded microbial genome coverage and improved protein family annotation in the COG database. Nucleic Acids Res..

[35] Cho BK, Kim D, Knight EM, Zengler K, Palsson BO (2014). Genome-scale reconstruction of the sigma factor network in Escherichia coli: topology and functional states. BMC Biol..

[36] O’Brien EJ, Monk JM, Palsson BO (2015). Using Genome-scale Models to Predict Biological Capabilities. Cell.

[37] Cho BK, Knight EM, Palsson BO (2006). PCR-based tandem epitope tagging system for Escherichia coli genome engineering. Biotechniques.

[38] Datta S, Costantino N, Court DL (2006). A set of recombineering plasmids for gram-negative bacteria. Gene.

[39] Langmead B, Trapnell C, Pop M, Salzberg SL (2009). Ultrafast and memory-efficient alignment of short DNA sequences to the human genome. Genome Biol..

[40] Wang L (2014). MACE: model based analysis of ChIP-exo. Nucleic Acids Res..

[41] Levin JZ (2010). Comprehensive comparative analysis of strand-specific RNA sequencing methods. Nat. Methods.

[42] Trapnell C (2010). Transcript assembly and quantification by RNA-Seq reveals unannotated transcripts and isoform switching during cell differentiation. Nat. Biotechnol..

[43] Aziz RK (2008). The RAST Server: rapid annotations using subsystems technology. BMC Genomics.

