# Supplementary material for: Revealing genome-scale transcriptional regulatory landscape of OmpR highlights its expanded regulatory roles under osmotic stress in Escherichia coli K-12 MG1655
Source: Sci Rep. 2017 May 19;7:2181. doi: 10.1038/s41598-017-02110-7 (PMC5438342; doi:10.1038/s41598-017-02110-7)
Supplement: Supplementary file 1 — Supplementary Information [file 41598_2017_2110_MOESM1_ESM.doc]

**Supplementary Information for:**

**Revealing genome-scale transcriptional regulatory landscape of OmpR highlights its expanded regulatory roles under osmotic stress in *Escherichia coli* K-12 MG1655**

Sang Woo Seo^1,2,8,*^, Ye Gao^3,8^, Donghyuk Kim^2,5,8^, Richard Szubin^2^, Jina Yang^1^, Byung-Kwan Cho^6,7^ and Bernhard O. Palsson^2,4,7,*^

^1^School of Chemical and Biological Engineering and Institute of Chemical Process, Seoul National University, 1 Gwanak-ro, Gwanak-Gu, Seoul 08826, Republic of Korea

^2^Department of Bioengineering, ^3^Division of Biological Science, and ^4^Department of Pediatrics, University of California San Diego, La Jolla, CA 92093, USA

^5^Department of Genetic Engineering, College of Life Sciences, Kyung Hee University, Yongin 446-701, Republic of Korea

^6^Department of Biological Sciences, Korea Advanced Institute of Science and Technology, Daejeon 305-701, Republic of Korea

^7^Novo Nordisk Foundation Center for Biosustainability, Technical University of Denmark, 2800 Lyngby, Denmark

^8^Co-first author.

^*^To whom correspondence.

Email: [palsson@ucsd.edu](mailto:palsson@ucsd.edu) (B.O.P); [swseo@snu.ac.kr](mailto:swseo@snu.ac.kr) (S.W.S)

**Supplementary Figures**


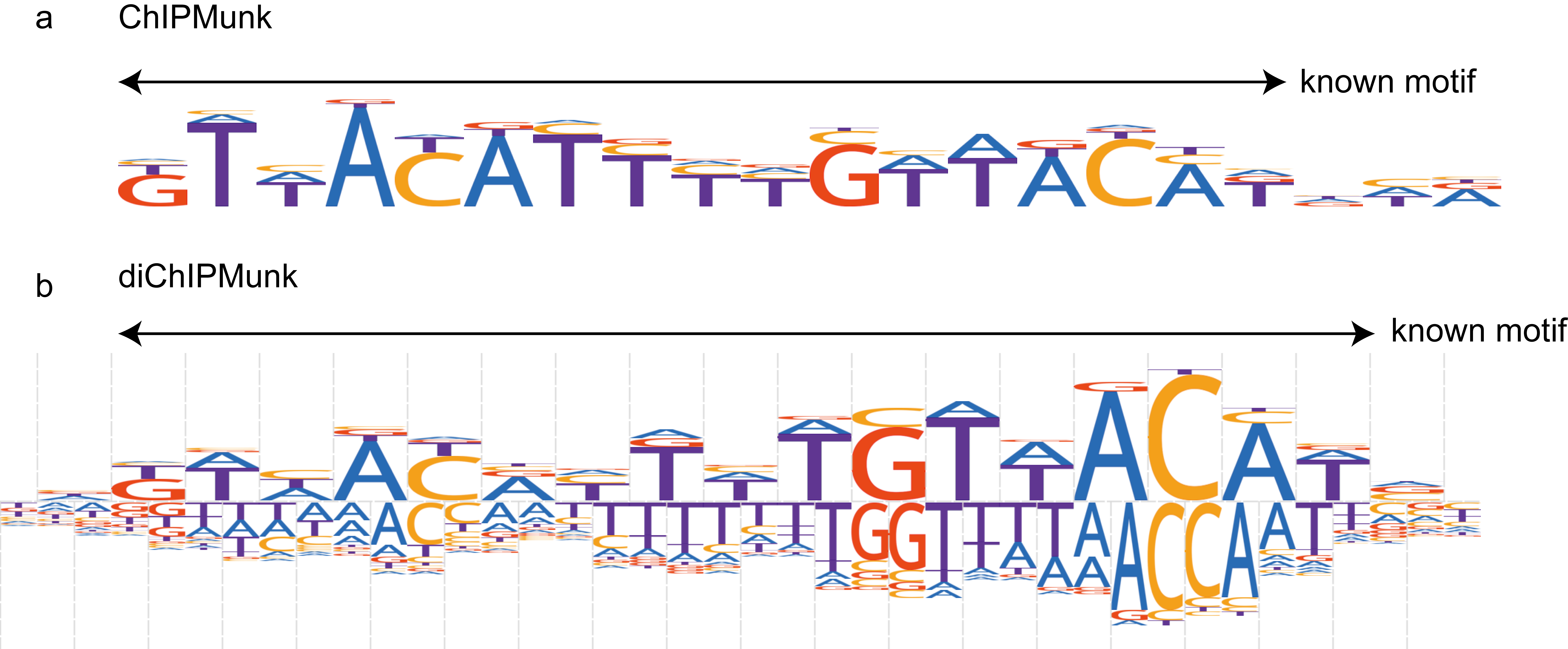


**Supplementary Figure 1. The OmpR binding motif sequences.** The motif sequences were generated by using (a) ChIPMunk and (b) diChIPMunk methods. Basically, both methods generated the previously known binding motif (GTTACATTTTGTTACAT).

**Supplementary Tables**

**Supplementary Table 1. OmpR-associated regions identified by ChIP-exo analysis.**

| **Binding**  **Sites** | **Transcription**  **Unit** | **Peak** | **ChIP-exo**  **Start** | **ChIP-exo**  **End** | **Causal relationship upon K/O** | **Regulatory**  **Mode** | **Distance**  **to TSS** | **S/N**  **ratio** | **Novel target?** | **Localization** |
| --- | --- | --- | --- | --- | --- | --- | --- | --- | --- | --- |
| 1 | *mraZ* | P1 | 89209 | 89268 | N | - | -350.5 | 6.86 | Y | Cytosol |
| 2 | *tomB* | P2 | 480063 | 480122 | N | - | -73.5 | 1.78 | Y | Cytosol |
| 3 | *copA* | P3 | 510775 | 510834 | Y | Rep | -169.5 | 2.51 | Y | IM |
| 4 | *nmpC* | P4 | 576106 | 576166 | Y | Rep | 43 | 2.26 | N | OM |
| 5 | *cstA* | P5 | 629059 | 629118 | N | - | 7.5 | 4.91 | Y | IM |
| 6 | *ompX* | P6 | 849553 | 849612 | N | - | -57.5 | 110.71 | Y | OM |
| 7 | *ompF* | P7 | 986373 | 986432 | Y | Act | -87.5 | 43.04 | N | OM |
| 8 | *yccT* | P8 | 1027037 | 1027089 | Y | Rep | -5 | 3.07 | Y | Cytosol |
| 9 | *csgDEFG* | P9 | 1102597 | 1102656 | Y | Act | - | 76.17 | N | IM-OM-OM-IM |
| 10 | *narUZYWV* | P10 | 1542224 | 1542283 | Y | Act | - | 14.83 | Y | IM-IM-IM-Cytosol-IM |
| 11 | *dtpA* | P11 | 1710621 | 1710680 | Y | Act | -46.5 | 11.01 | N | IM |
| 12 | *htpX* | P12 | 1910702 | 1910754 | Y | Rep | -86 | 1.89 | Y | IM |
| 13 | *yobB-exoX* | P13 | 1923367 | 1923419 | Y | Rep | -62 | 2.01 | Y | Cytosol-Cytosol |
| 14 | *znuA* | P14 | 1940588 | 1940647 | Y | Act | - | 2.46 | Y | IM |
| 15 |  | P15 | 1940818 | 1940875 | Y | Act | - | 2.23 | Y |  |
| 16 | *flhDC* | P16 | 1976353 | 1976412 | N | - | - | 19.75 | Y | Cytosol-Cytosol |
| 17 | *yedS_1-yedS_2* | P17 | 2031915 | 2031974 | N | - | - | 20.52 | Y | - |
| 18 | *ompC/micF* | P18 | 2310906 | 2310960 | Y | Act | -83 | 64.94 | N | OM |
| 19 | *nuoN* | P19 | 2389715 | 2389774 | Y | Rep | 47.5 | 3.21 | Y | IM |
| 20 | *fadL* | P20 | 2459263 | 2459322 | N | - | 65.5 | 2.24 | N | OM |
| 21 | *queE* | P21 | 2903494 | 2903554 | Y | Act | 589 | 33.82 | Y | Cytosol |
| 22 | *galP* | P22 | 3086060 | 3086119 | Y | Rep | -187.5 | 5.33 | Y | IM |
| 23 | *alx* | P23 | 3236367 | 3236426 | Y | Rep | -100.5 | 18.87 | Y | IM |
| 24 | *sstT* | P24 | 3237874 | 3237933 | N | - | 18.5 | 6.91 | Y | IM |
| 25 | *malEFG* | P25 | 4244765 | 4244851 | Y | Rep | -321 | 1.86 | Y | IM-IM-IM |

**Supplementary Table 2. Confirmation of previously characterized OmpR-binding sites**

| **Number** | **Transcription Unit** | **Decision** |
| --- | --- | --- |
| 1 | *bolA* | No |
| 2 | *nmpC* | Yes |
| 3 | *ompF* | Yes |
| 4 | *csgDEFG* | Yes |
| 5 | *tppB* | Yes |
| 6 | *ompC* | Yes |
| 7 | *micF* | Yes |
| 8 | *fadL* | Yes |
| Total | | 7/8 (88%) |

**Supplementary Table 3. Other transcription factors known to regulate OmpR regulons**.

| **TFs** | **# of bindings** | **Target genes** |
| --- | --- | --- |
| IHF | 8 | *nmpC/ompC/csgG/micF/csgE/csgD/nuoN/csgF* |
| CRP | 8 | *nmpC/cstA/flhC/flhD/malF/malG/malE/fadL* |
| CpxR | 7 | *ompF/ompC/csgG/csgF/tomB/csgD/csgE* |
| FliZ | 6 | *flhC/flhD/csgG/csgF/csgE/csgD* |
| CsgD | 5 | *csgG/yccT/csgE/csgD/csgF* |
| BasR | 5 | *csgG/csgF/tomB/csgD/csgE* |
| Cra | 4 | *csgG/csgF/csgE/csgD* |
| MlrA | 4 | *csgG/csgF/csgE/csgD* |
| RcdA | 4 | *csgG/csgF/csgE/csgD* |
| RstA | 4 | *csgG/csgF/csgE/csgD* |
| MalT | 3 | *malF/malG/malE* |
| H-NS | 2 | *flhC/flhD* |
| MatA | 2 | *flhC/flhD* |
| ArcA | 2 | *nuoN/fadL* |
| RcsAB | 2 | *flhC/flhD* |
| LrhA | 2 | *flhC/flhD* |
| Lrp | 2 | *micF/ompC* |
| HdfR | 2 | *flhC/flhD* |
| Zur | 1 | *znuA* |
| FadR | 1 | *fadL* |
| Rob | 1 | *micF* |
| SoxS | 1 | *micF* |
| PdhR | 1 | *mraZ* |
| CueR | 1 | *copA* |
| GalS | 1 | *galP* |
| GalR | 1 | *galP* |
| MarA | 1 | *micF* |
| NagC | 1 | *galP* |
| FNR | 1 | *nuoN* |
| Fis | 1 | *nuoN* |

**Supplementary Table 4. Primers used in this study.**

| **Primers** | **Sequence (5’-3’)** |
| --- | --- |
| copA_KO_Forward | TTTATCACAGCCAGTCAAAACTGTCTTAAAGGAGTGTTTTATGTCACAAACTATCGACCTATTCCGGGGATCCGTCGACC |
| copA_KO_Reverse | AAATGCGCCACCCTAAAGCAGCGCATCCGCAATGATGTACTTATTCCTTCGGTTTAAACCGTGTAGGCTGGAGCTGCTTC |
| copA_KO_Confirm_For | GACTTTTACCCGCCTGGTTT |
| copA_KO_Confirm_Rev | AACCTGTGCCTGAACCGTAG |
| htpX_KO_Forward | CGCATATTGCGTTTTGTTAAACTGAGGTAAAAAGAAAATTATGATGCGAATCGCGCTCTTATTCCGGGGATCCGTCGACC |
| htpX_KO_Reverse | GCGCGTCGATCAGGACGCGCTTTTTAGTATTTACTTCATATTACTTCAGGTATTCACCCGGTGTAGGCTGGAGCTGCTTC |
| htpX_KO_Confirm_For | AGCGACAAGATGCCGTAAAT |
| htpX_KO_Confirm_Rev | TATCACTCAGCCACGATCCA |
| yobB_KO_Forward | CGCACTCATAATTTGCAGTCATTTTGAAAAGGAAGTCATTATGTCGTTCTGGAAAGTTGCATTCCGGGGATCCGTCGACC |
| yobB_KO_Reverse | TCGATAATGCGCAACATGGCATGCTCCAGGCCGAAAAAGCCTAGCGTAATGGAATGATATGTGTAGGCTGGAGCTGCTTC |
| yobB_KO_Confirm_For | TGAACGTGAACAGCCTGAAC |
| yobB_KO_Confirm_Rev | TCATGGGGTTGACGATTTTT |
| nuoN_KO_Forward | GTTTGTTAATTCCGTTACTACTACAAGGCCGTAAATCGCCATGACAATAACTCCACAAAAATTCCGGGGATCCGTCGACC |
| nuoN_KO_Reverse | CCGAGCATGTCGGCGGCTTTCTGACTTACAAAGTAACAGATTACATCAGCGGCATTGCCAGTGTAGGCTGGAGCTGCTTC |
| nuoN_KO_Confirm_For | GCTGCGTGAGCTGTTTATGA |
| nuoN_KO_Confirm_Rev | GGGCAAAATTCAGGCAGTTA |
| galP_KO_Forward | TCTTAATTCACAATAAAAAATAACCATATTGGAGGGCATCATGCCTGACGCTAAAAAACAATTCCGGGGATCCGTCGACC |
| galP_KO_Reverse | AGAGGTGGCTTCCTCCGCGATGGGAGGAAGCTTGGGGAGATTAATCGTGAGCGCCTATTTGTGTAGGCTGGAGCTGCTTC |
| galP_KO_Confirm_For | AACCATATTGGAGGGCATCA |
| galP_KO_Confirm_Rev | AGTTTTGGCTCCGGGTAGTT |
| alx_KO_Forward | CTTCCGACGTTGGCCGTTTTTTTATGTGTAAGGAACTTCTATGAATACTGTCGGCACGCCATTCCGGGGATCCGTCGACC |
| alx_KO_Reverse | CATGTTAATGACAGGGTACACGGCTTAGGCAGATTAAAAATTATCCACCCCGCTGCTTATGTGTAGGCTGGAGCTGCTTC |
| alx_KO_Confirm_For | CAACCCGAAAGGAATACGC |
| alx_KO_Confirm_Rev | TGCGAAAAATTACCCTCACC |
| malE_KO_Forward | GGTGTTTTCACGAGCACTTCACCAACAAGGACCATAGATTATGAAAATAAAAACAGGTGCATTCCGGGGATCCGTCGACC |
| malE_KO_Reverse | CCGGACAAGGCGTTCACGCCGCATCCGGCATTTCACAGCATTACTTGGTGATACGAGTCTGTGTAGGCTGGAGCTGCTTC |
| malE_KO_Confirm_For | ATGTGCGCATCTCCACATTA |
| malE_KO_Confirm_Rev | ACGCCTTATCCGACAACAAC |
| znuA_KO_Forward | TATCACACTTCTCATATTCATTACGATTATTGGTCGCATTATGTTACATAAAAAAACGCTATTCCGGGGATCCGTCGACC |
| znuA_KO_Reverse | CGACAGAGCGGGCTATCTGTTGCACGTAATCACTTCCTCATTAATCTCCTTTCAGGCAGCGTGTAGGCTGGAGCTGCTTC |
| znuA_KO_Confirm_For | TTCCAGGGAAACCAGACTTG |
| znuA_KO_Confirm_Rev | TAAACATAAGGCCGCCAGAC |
| narU_KO_Forward | GTCTTTTTTTCATTACCAATGTGTGCATGTGAGGAACAATATGGCACTGCAAAATGAGAAATTCCGGGGATCCGTCGACC |
| narU_KO_Reverse | TTGTCGGATGCGACACGGTTGTACATCAGGCATCGATCTCTTATTTTTGGCTGAACTTCCGTGTAGGCTGGAGCTGCTTC |
| narU_KO_Confirm_For | CGCGGTTACGCTTTCTTATC |
| narU_KO_Confirm_Rev | AGTAGCGAAAGCGATCCAAA |
| tppB_KO_Forward | AGCACCCCCGTTAATATGGGATGTAAAAAAAGAGGTAAAAGTGTCCACTGCAAACCAAAAATTCCGGGGATCCGTCGACC |
| tppB_KO_Reverse | AACGAAAAGTTAGCGGCTTGTAAAAGAGTTTCCCTGAATTTTACGCTACGGCTGCTTTCGGTGTAGGCTGGAGCTGCTTC |
| tppB_KO_Confirm_For | TTCCCCGCCTCAGTTATATG |
| tppB_KO_Confirm_Rev | TTTTACGCTACGGCTGCTTT |
| tomb_KO_Forward | AAGGTGTCCGTTAGTTCAACCGCTAAGAAGGGGACGCGTTATGGATGAATACTCACCCAAATTCCGGGGATCCGTCGACC |
| tomb_KO_Reverse | AGGTTTTTCGGACATACTTCTACCTATGGTTGTAATAATTCTAACAAGATAAACTCGCAGGTGTAGGCTGGAGCTGCTTC |
| tomb_KO_Confirm_For | GGGCAAGTCCAGGTCAGTAA |
| tomb_KO_Confirm_Rev | GTGTCAATTGTCTGGCAACG |
| ompF_KO_Forward | GTGGCAGGTGTCATAAAAAAAACCATGAGGGTAATAAATAATGATGAAGCGCAATATTCTATTCCGGGGATCCGTCGACC |
| ompF_KO_Reverse | AAAGTCCTGTTTTTTCGGCATTTAACAAAGAGGTGTGCTATTAGAACTGGTAAACGATACGTGTAGGCTGGAGCTGCTTC |
| ompF_KO_Confirm_For | ACTTTCACGGTAGCGAAACG |
| ompF_KO_Confirm_Rev | TTTGCAAGACGTGAGATTGC |
| ygcF_KO_Forward | TCTGCAATAATTAACAATTATCCACTTCACAGAGAATGCTATGCAGTACCCGATTAACGAATTCCGGGGATCCGTCGACC |
| ygcF_KO_Reverse | TTACAGGTTCCACCCATTTATAACGCTTATAAATGTTTAATCAGGCAATATTTAGATATTGTGTAGGCTGGAGCTGCTTC |
| ygcF_KO_Confirm_For | ACATGATTGTTGTGGCAGGA |
| ygcF_KO_Confirm_Rev | AGGAGTTTTCAGAGGCGACA |
| ompX_KO_Forward | TTAAAACTTAGGACTTATTTGAATCACATTTGAGGTGGTTATGAAAAAAATTGCATGTCTATTCCGGGGATCCGTCGACC |
| ompX_KO_Reverse | ATCCGCCCCGAGAGGCGGATTTTTTATATCACCAAAGTGATTAGAAGCGGTAACCAACACGTGTAGGCTGGAGCTGCTTC |
| ompX_KO_Confirm_For | AATGGCCCGTTAGACATCAC |
| ompX_KO_Confirm_Rev | TCGGAACCGATATTTTCGAC |
| yccT_KO_Forward | ACACATTATTATTCGGGCGGTCTTTTGACAGGAGCTACCTATGAAAACCGGCATCGTGACATTCCGGGGATCCGTCGACC |
| yccT_KO_Reverse | AGATAAAGACCGAAAAAGCCTGCGCACAGGCACAAAAATCTCAGGAAGATGGTTGTTTTTGTGTAGGCTGGAGCTGCTTC |
| yccT_KO_Confirm_For | ATGCCTGCAATATCGGTTTC |
| yccT_KO_Confirm_Rev | GCACAGGCACAAAAATCTCA |
| copA_OE_Forward | CACCATGTCACAAACTATCGAC |
| copA_OE_Reverse | TTCCTTCGGTTTAAACCGCAGC |
| htpX_OE_Forward | CACCATGATGCGAATCGCGCTC |
| htpX_OE_Reverse | CTTCAGGTATTCACCCGTACGC |
| yobB_OE_Forward | CACCATGTCGTTCTGGAAAGTT |
| yobB_OE_Reverse | GCGTAATGGAATGATATCGCCT |
| nuoN_OE_Forward | CACCATGACAATAACTCCACAA |
| nuoN_OE_Reverse | AAACATCAGCGGCATTGCCAAA |
| psrN-alx_OE_Forwrad | CACCGCAAAGGGGAGTAACTTC |
| psrN-alx_OE_Reverse | TCCACCCCGCTGCTTATCATGC |
| narU_OE_Forward | CACCATGGCACTGCAAAATGAG |
| narU_OE_Reverse | TTTTTGGCTGAACTTCCGCCGA |
| ompF_OE_Forward | CACCATGATGAAGCGCAATATT |
| ompF_OE_Reverse | GAACTGGTAAACGATACCCACA |
| yccT_OE_Forward | CACCATGAAAACCGGCATCGTG |
| yccT_OE_Reverse | GGAAGATGGTTGTTTTTCCGCC |
| ompX_OE_Forward | CACCATGAAAAAAATTGCATGT |
| ompX_OE_Reverse | GAAGCGGTAACCAACACCGGCA |
